# Establishment of a synchronized tyrosinase transport system revealed a role of Tyrp1 in efficient melanogenesis by promoting tyrosinase targeting to melanosomes

**DOI:** 10.1038/s41598-024-53072-6

**Published:** 2024-01-30

**Authors:** Hikari Nakamura, Mitsunori Fukuda

**Affiliations:** Laboratory of Membrane Trafficking Mechanisms, Department of Integrative Life Sciences, Graduate School of Life Sciences, Tohoku University, Aobayama, Aoba-Ku, Sendai, Miyagi 980-8578 Japan

**Keywords:** Membrane trafficking, Organelles, Protein transport

## Abstract

Tyrosinase (Tyr) is a key enzyme in the process of melanin synthesis that occurs exclusively within specialized organelles called melanosomes in melanocytes. Tyr is synthesized and post-translationally modified independently of the formation of melanosome precursors and then transported to immature melanosomes by a series of membrane trafficking events that includes endoplasmic reticulum (ER)-to-Golgi transport, post-Golgi trafficking, and endosomal transport. Although several important regulators of Tyr transport have been identified, their precise role in each Tyr transport event is not fully understood, because Tyr is present in several melanocyte organelles under steady-state conditions, thereby precluding the possibility of determining where Tyr is being transported at any given moment. In this study, we established a novel synchronized Tyr transport system in *Tyr*-knockout B16-F1 cells by using Tyr tagged with an artificial oligomerization domain FM4 (named Tyr-EGFP-FM4). Tyr-EGFP-FM4 was initially trapped at the ER under oligomerized conditions, but at 30 min after chemical dissociation into monomers, it was transported to the Golgi and at 9 h reached immature melanosomes. Melanin was then detected at 12 h after the ER exit of Tyr-EGFP-FM4. By using this synchronized Tyr transport system, we were able to demonstrate that Tyr-related protein 1 (Tyrp1), another melanogenic enzyme, is a positive regulator of efficient Tyr targeting to immature melanosomes. Thus, the synchronized Tyr transport system should serve as a useful tool for analyzing the molecular mechanism of each Tyr transport event in melanocytes as well as in the search for new drugs or cosmetics that artificially regulate Tyr transport.

## Introduction

Tyrosinase (Tyr) is a typical type I integral membrane protein that localizes at melanosomes in melanocytes^1^, and it serves as a rate-limiting enzyme in the process of melanin synthesis in melanosomes^2^. Since mutations in the *Tyr* gene cause a genetic pigmentation disorder called albinism in mammals, e.g., human oculocutaneous albinism type 1 (OCA1)^3^, Tyr is generally recognized as a critical factor in producing skin and hair pigmentation in mammals, specifically in accounting for the differences in skin color among human races^4^. Although the melanin whose synthesis is catalyzed by Tyr is crucial in achieving photoprotection, abnormal melanin accumulation causes hyperpigmentation disorders such as melasma, senile lentigo, and freckles^5^. Thus, inhibiting the enzymatic activity of Tyr has often been used as a major strategy for skin whitening in the cosmetic industry^6^.

Melanosomes are formed and mature in a stepwise fashion and they are morphologically classified into four stages (from I to IV)^7,8^. Stage I and stage II melanosomes are transparent (hence referred to as immature melanosomes or premelanosomes) because of the absence of melanogenic enzymes such as Tyr and of melanin. Tyr is independently synthesized and transported to immature melanosomes by a series of membrane trafficking events that lead to melanin synthesis in stage III and stage IV melanosomes. After protein translation, Tyr is first inserted into the endoplasmic reticulum (ER), then transported to the Golgi, where Tyr is highly glycosylated, and subsequently to immature melanosomes via endosomes^9–11^. Several important regulators of Tyr transport have been identified by genetic and biochemical analyses of the gene products responsible for genetic pigmentation disorders such as Hermansky-Pudlak syndrome (HPS)^9,12^. For example, HPS1 and HPS4 have been identified as a heterodimeric activator (known as biogenesis of lysosome-related organelles complex-3, BLOC-3) for the small GTPases Rab32 and Rab38 (Rab32/38), both of which are involved in Tyr transport from the Golgi^13–16^. Moreover, these Rabs have also been reported to regulate the recycling of VAMP7 from melanosomes^17^ and large dense core vesicles (another type of lysosome-related organelles) in intestinal Paneth cells^18^. Although important regulators of Tyr transport have been identified, their precise role in each Tyr transport event (e.g., ER-to-Golgi transport, transport from the Golgi, and endosomal transport) is not completely understood.

A major obstacle in attempts to analyze each Tyr transport event has been the fact that under steady-state conditions Tyr is present in several melanocyte organelles: Tyr is often localized in the perinuclear region (mostly at the Golgi) in addition to being localized at immature and mature melanosomes. Thus, it is extremely difficult to determine where Tyr is being transported at any given moment even when Tyr has been fluorescently tagged in living cells. To overcome this obstacle, in the present study, we established a novel synchronized Tyr transport system by using an artificial oligomerization domain (i.e., FKBP12-derived FM4 domain)^19^. Using the synchronized transport system enabled us to demonstrate that tyrosinase-related protein 1 (Tyrp1), another melanogenic enzyme, promotes efficient targeting of Tyr to melanosomes and efficient melanogenesis. Based on our findings, we discuss the utility of this tool for identifying regulators of each Tyr transport event as well as in the search for new drugs or cosmetics that artificially promote or inhibit Tyr transport to melanosomes.

## Results

### Tyr-EGFP was functional in melanin synthesis in *Tyr*-KO B16F1 cells

**Figure 1:**
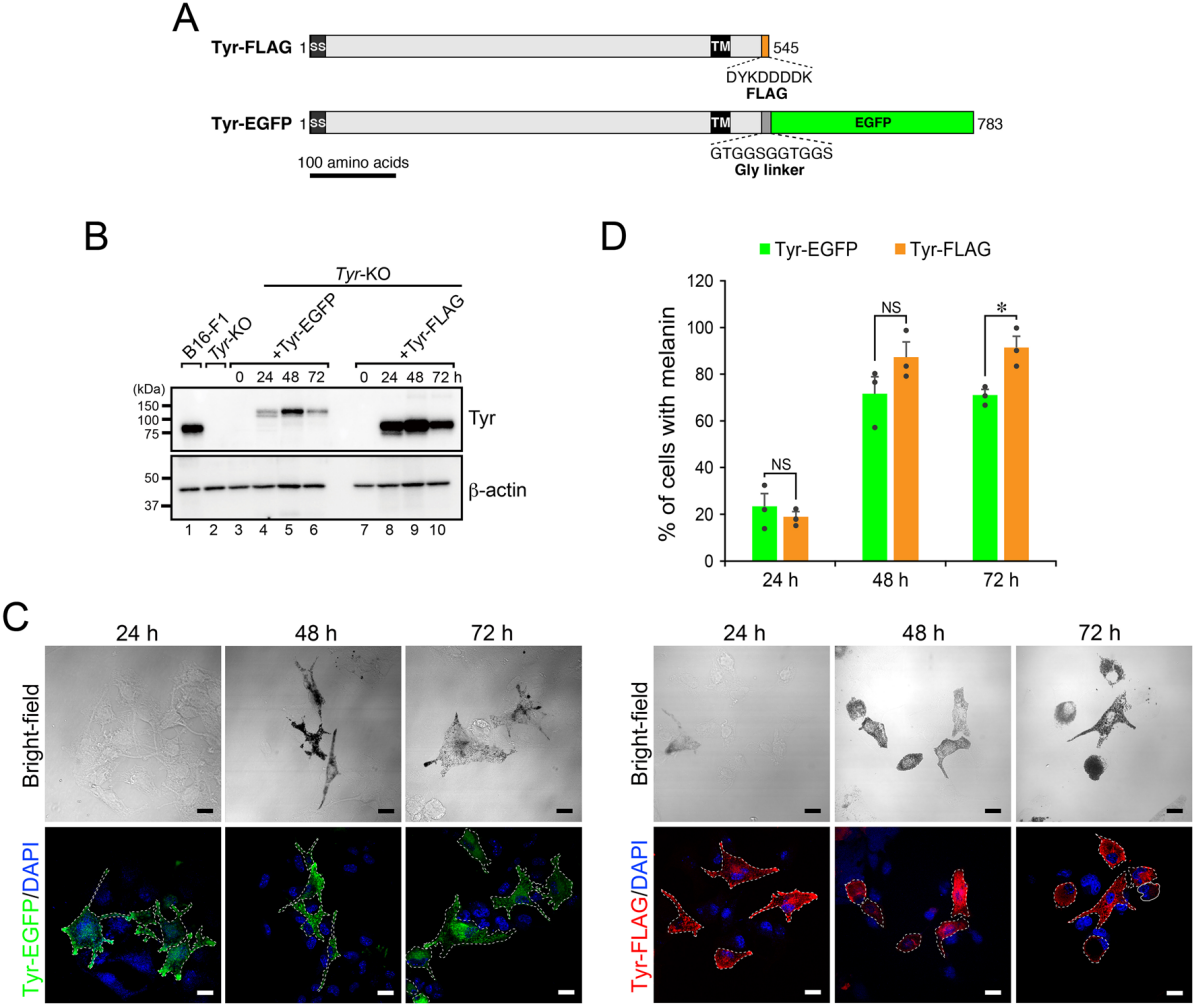
EGFP-tagged Tyr was functional in *Tyr*-KO B16-F1 cells. (**A**) Schematic representation of mouse Tyr C-terminal tagged with FLAG (Tyr-FLAG) and EGFP (Tyr-EGFP). SS, signal sequence; TM, transmembrane domain. (**B**) Expression of Tyr-EGFP in *Tyr*-KO cells. After plasmid transfection, *Tyr*-KO B16-F1 cells expressing Tyr-EGFP or Tyr-FLAG were lysed at the times indicated and analyzed by immunoblotting with anti-Tyr and anti-β-actin antibodies. WT and *Tyr*-KO cells were used as a positive control and a negative control, respectively, for Tyr expression. (**C**) Representative images of Tyr-EGFP-expressing and Tyr-FLAG-expressing *Tyr*-KO cells after plasmid transfection. Scale bars, 20 μm. (**D**) The percentage of *Tyr*-KO cells expressing Tyr-EGFP or Tyr-FLAG and containing melanin shown in (**C**) was calculated at the times indicated after plasmid transfection. The error bars represent the means ± SEM of the data obtained in three independent experiments (n = 30 cells in each experiment). ∗ *P* < 0.05; NS, not significant (one-way ANOVA and Tukey’s test). Only the statistical significance between Tyr-EGFP and Tyr-FLAG at each time point was shown.

To visualize Tyr in melanocytes, we generated mouse Tyr C-terminal tagged with enhanced green fluorescent protein (EGFP), in which the Tyr and EGFP were separated by a short Gly linker (simply referred to as Tyr-EGFP hereafter) to prevent any possible steric hindrance to Tyr sorting and transport (Fig. 1A). To investigate whether the fluorescence-tagged Tyr functioned in melanin synthesis, we transiently expressed Tyr-EGFP (or Tyr-FLAG^20^) in melanin-deficient *Tyr*-KO B16-F1 cells^21^ and evaluated its expression level by immunoblotting and melanin synthesis by light microscopy. We used Tyr with a short FLAG tag (i.e., 8 amino acids) as a positive control, because we previously showed that Tyr-FLAG fully restored melanin synthesis in Tyr-deficient melanocytes^20^. As shown in Fig. 1B, Tyr-EGFP expression was first observed at 24 h after the transfection, peaked at 48 h, and then decreased to the 24-h-level at 72 h, the same as Tyr-FLAG expression did. However, the total expression level of Tyr-EGFP was clearly lower than that of Tyr-FLAG, suggesting that EGFP-tagging affects protein folding, stability, and/or degradation. Despite the lower expression level of Tyr-EGFP, it was capable of rescuing melanin synthesis in *Tyr*-KO cells at 48 h after transfection, although EGFP fluorescence in the peripheral area of the cells was first observed at 24 h (Fig. 1C). The results of a quantitative analysis indicated that ~ 70% of the Tyr-EGFP-expressing cells contained black melanin, which was almost the same as in the Tyr-FLAG-expressing cells (~ 80%) (Fig. 1D). The time course of melanin recovery in the Tyr-EGFP-expressing cells and Tyr-FLAG-expressing cells was also almost the same (Fig. 1D), suggesting that Tyr-EGFP is most likely transported to melanosomes, the same as Tyr-FLAG is. These results taken together indicated that Tyr-EGFP functions in melanin synthesis in *Tyr*-KO cells.

### Establishment of a synchronized Tyr transport system

**Figure 2:**
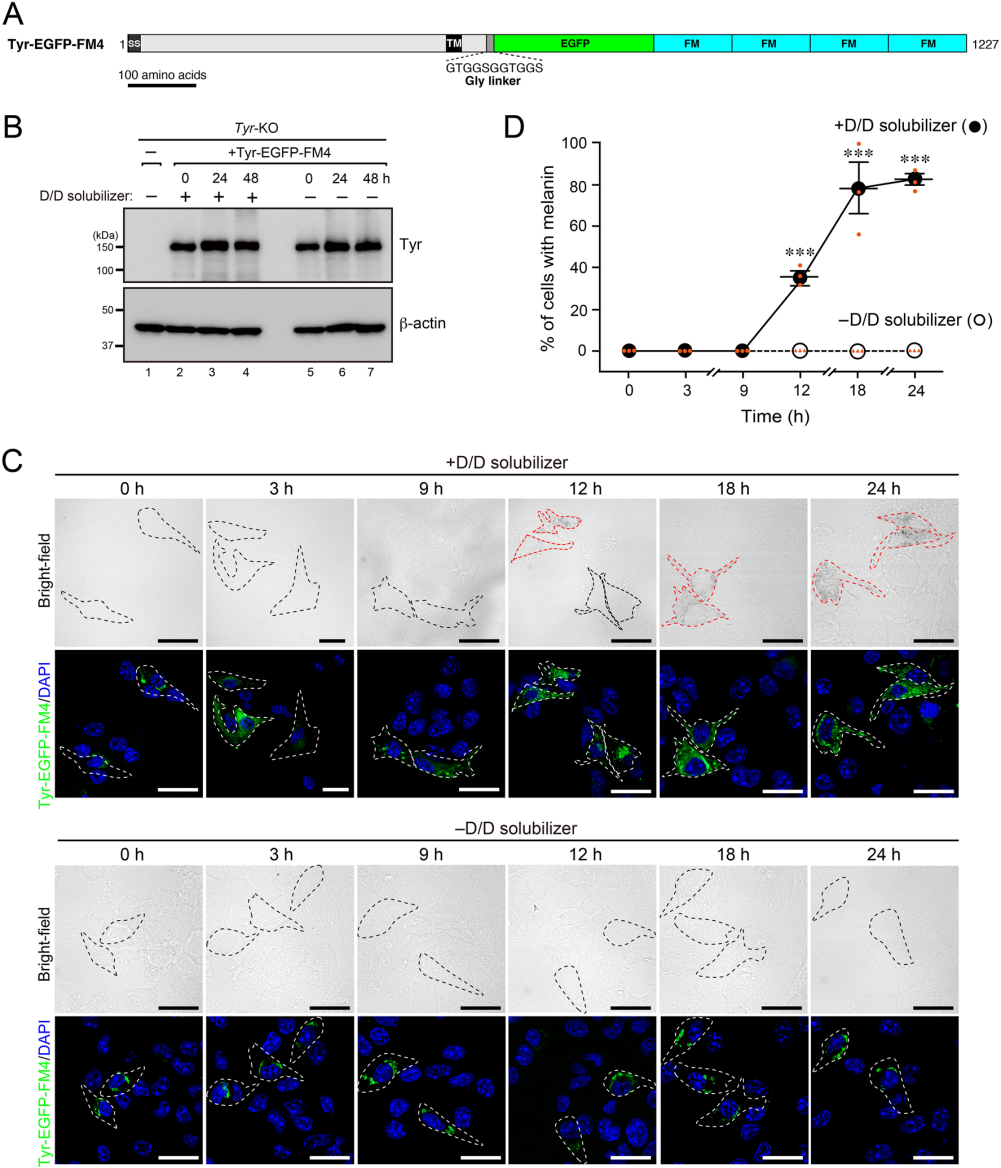
Establishment of a synchronized Tyr transport system by using the FM4-mediated oligomerization system. (**A**) Schematic representation of mouse Tyr C-terminal tagged with EGFP and four FM domains (Tyr-EGFP-FM4). FM is a FKBP12-derived artificial oligomerization domain^19^. (**B**) Expression of Tyr-EGFP-FM4 in the presence and absence of D/D solubilizer. *Tyr*-KO B16-F1 cells expressing Tyr-EGFP-FM4 were treated or not treated with 500 nM D/D solubilizer (at time 0). The cells were then harvested at the times indicated and analyzed by immunoblotting with anti-Tyr and anti-β-actin antibodies. (**C**) Representative images of Tyr-EGFP-FM4-expressing *Tyr*-KO cells after treatment with D/D solubilizer or DMSO (–D/D solubilizer). Tyr-EGFP-FM4-expressing cells in the fluorescent images are outlined with broken white lines. Melanin-containing Tyr-EGFP-FM4-expressing cells and transparent Tyr-EGFP-FM4-expressing cells in the bright-field images are outlined with broken red lines and broken black lines, respectively. Scale bars, 20 μm. (**D**) The percentage of cells shown in (**C**) containing melanin was calculated after D/D solubilizer treatment (closed circles) or DMSO treatment (–D/D solubilizer; open circles). The error bars represent the means ± SEM of the data obtained in three independent experiments (n = 30 cells in each experiment). ∗ ∗ ∗ *P* < 0.001 (one-way ANOVA and Tukey’s test).

To synchronize Tyr transport to melanosomes, we turned our attention to four FKBP12-derived artificial oligomerization domains (named FM4)^19^ and attempted to trap Tyr-EGFP at the ER. FM4 fusion proteins in the secretory pathway are known to form large aggregates at the ER and to be trapped there. D/D solubilizer, a commercially available, membrane permeable ligand of FM4, dissolves the aggregates into a monomer and thereby initiates protein transport from the ER to the Golgi synchronously. We generated Tyr-EGFP-FM4 (Fig. 2A) and transiently expressed it in *Tyr*-KO B16-F1 cells. Although the protein expression level of Tyr-EGFP-FM4 was unchanged for 48 h regardless of whether D/D solubilizer was present or absent (Fig. 2B), black melanosomes were observed only in the presence of D/D solubilizer (Fig. 2C). Microscopic analysis revealed large green Tyr-EGFP-FM4 aggregates in the absence of D/D solubilizer, but no melanin-containing cells were observed (Fig. 2C, cells outlined with broken black lines in the lower panels), suggesting that Tyr-EGFP-FM4 continues to be trapped in the ER. By contrast, Tyr-EGFP-FM4 signals were dispersed into the cytoplasm at 3 h after D/D solubilizer treatment, and melanin was observed at 12 h (Fig. 2C, cells outlined with broken red lines in the upper panels). The results of the quantitative analysis of the melanin-containing cells indicated that ~ 40% of the Tyr-EGFP-FM4-expressing cells contained melanin at 12 h after D/D solubilizer treatment, and ~ 80% of the cells were recovered at 18 h (Fig. 2D).

**Figure 3:**
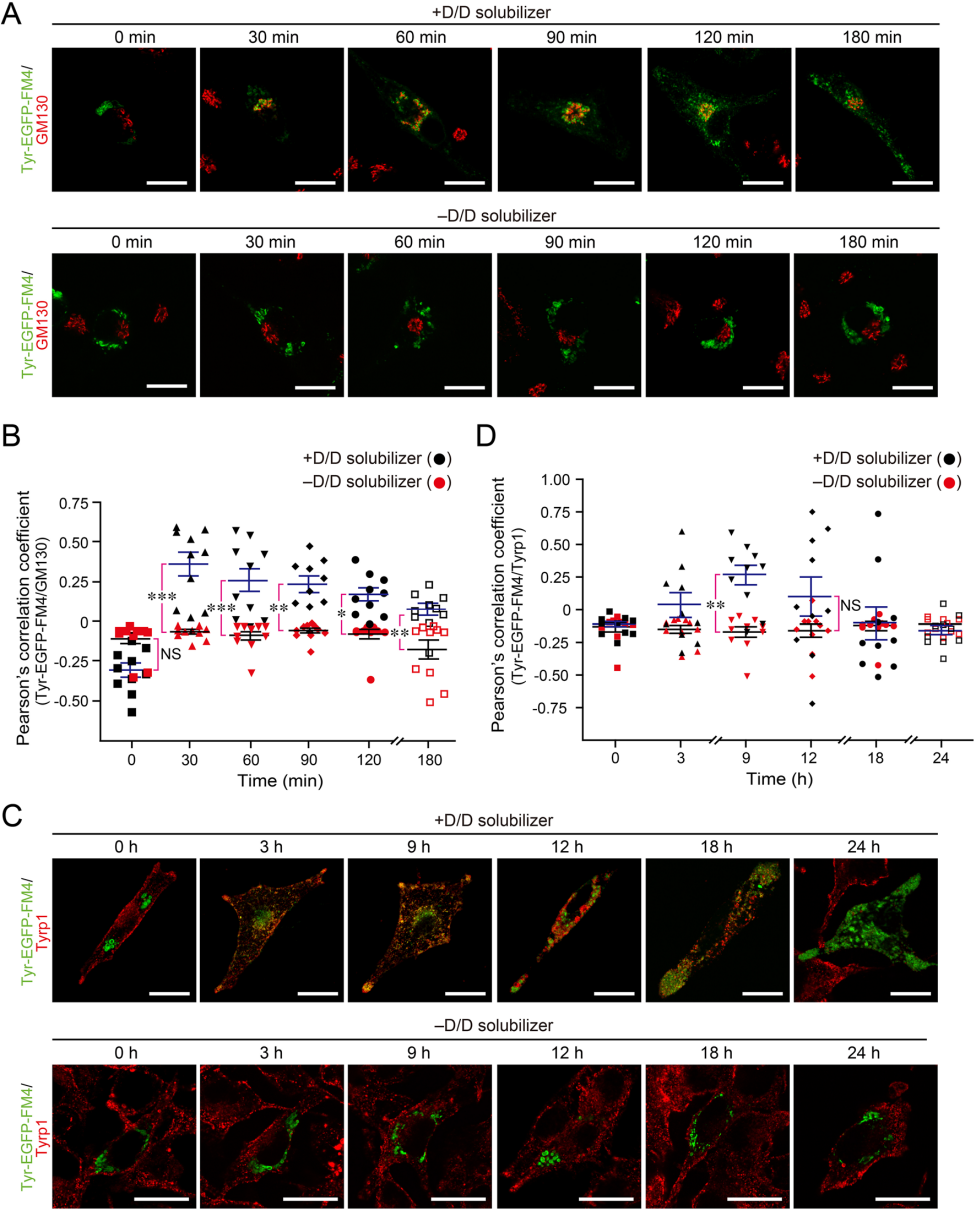
Colocalization of Tyr-EGFP-FM4 with GM130 at the Golgi and with Tyrp1 at melanosomes. (**A**) *Tyr*-KO B16-F1 cells expressing Tyr-EGFP-FM4 were treated with 500 nM D/D solubilizer or DMSO (–D/D solubilizer), fixed at the times indicated, and stained for GM130 (a Golgi marker). Scale bars, 20 μm. (**B**) Quantification of the colocalization ratio between Tyr and GM130 in the cells shown in (**A**) in the presence (black symbols) or absence of D/D solubilizer (red symbols). (**C**) *Tyr*-KO cells expressing Tyr-EGFP-FM4 were treated with 500 nM D/D solubilizer or DMSO (–D/D solubilizer), fixed at the times indicated, and stained for Tyrp1. Scale bars, 20 μm. (**D**) Quantification of the colocalization ratio between Tyr and Tyrp1 in the cells shown in (**C**) in the presence (black symbols) or absence of D/D solubilizer (red symbols). Pearson’s correlation coefficients in (**B** and **D**) were calculated (n = 10 cells), and the error bars represent the means ± SEM. ∗ *P* < 0.05; ∗ ∗ *P* < 0.01; ∗ ∗ ∗ *P* < 0.001; NS, not significant (one-way ANOVA and Tukey’s test). Only the statistical significance between the presence and absence of D/D solubilizer at each time point was shown.

To identify the time of the Tyr transport from the ER to the Golgi and to immature melanosomes, we compared the localization of Tyr-EGFP-FM4 and several organelle markers in *Tyr*-KO cells after D/D solubilizer treatment. First, we stained Golgi marker GM130 and examined the cells for colocalization between Tyr-EGFP-FM4 and GM130 (Fig. 3A). Their colocalization was first noted at 30 min after D/D solubilizer treatment and was still evident at 90 min (Fig. 3A, upper panels). By contrast, no colocalization between Tyr-EGFP-FM4 and GM130 was observed in the absence of D/D solubilizer (Fig. 3A, lower panels). Quantification of the colocalization rate between Tyr-EGFP-FM4 and GM130 in the cells shown in Fig. 3A revealed that the rate peaked at 30 min and then gradually decreased to the background level at 180 min (Fig. 3B). Thus, Tyr is likely to be transported from the ER to the Golgi for 30 min, which corresponds to the time of the ER-to-Golgi transport of model secretory proteins^22,23^. Moreover, their colocalization rate was always significantly higher in the presence of D/D solubilizer than in its absence at every time point, suggesting that some portion of the Tyr was always retained at the Golgi. Consistent with our observations, under steady-state conditions Tyr was distributed in the perinuclear region in addition to the peripheral melanosomes in the wild-type (WT) B16-F1 cells^21^.

Next, we stained melanosome-resident protein Tyrp1 in order to determine when Tyr-EGFP-FM4 reaches immature melanosomes in *Tyr*-KO cells (Fig. 3C). In the absence of D/D solubilizer, Tyrp1 was distributed in the periphery, and no colocalization between Tyr-EGFP-FM4 and Tyrp1 was observed (Fig. 3C, lower panels). Peripheral Tyrp1 is likely to be present at immature melanosomes, because it partially colocalized with premelanosomal fibril protein PMEL^24^ (Fig. S1A, arrowheads). By contrast, after D/D solubilizer treatment, Tyr-EGFP-FM4 was transported to the peripheral area and colocalized with Tyrp1 there at 9 h (Fig. 3C, upper panels), before melanin was first detected (see Fig. 2D, 12 h). Intriguingly, however, the Tyrp1 signals mostly disappeared at 18 h after D/D solubilizer treatment, and no signals were observed at 24 h, presumably because antibody access to Tyrp1 is inhibited by melanin deposition (Fig. S1B). Although the Tyrp1 signals gradually disappeared as melanin deposition proceeded under our experimental conditions, significant colocalization between Tyr-EGFP-FM4 and Tyrp1 was observed at 9 h after D/D solubilizer treatment (Fig. 3D), suggesting that Tyr reaches immature melanosomes at 9 h after it exits from the ER.

### Overexpression of Tyrp1 promoted Tyr transport to melanosomes and melanin synthesis

**Figure 4:**
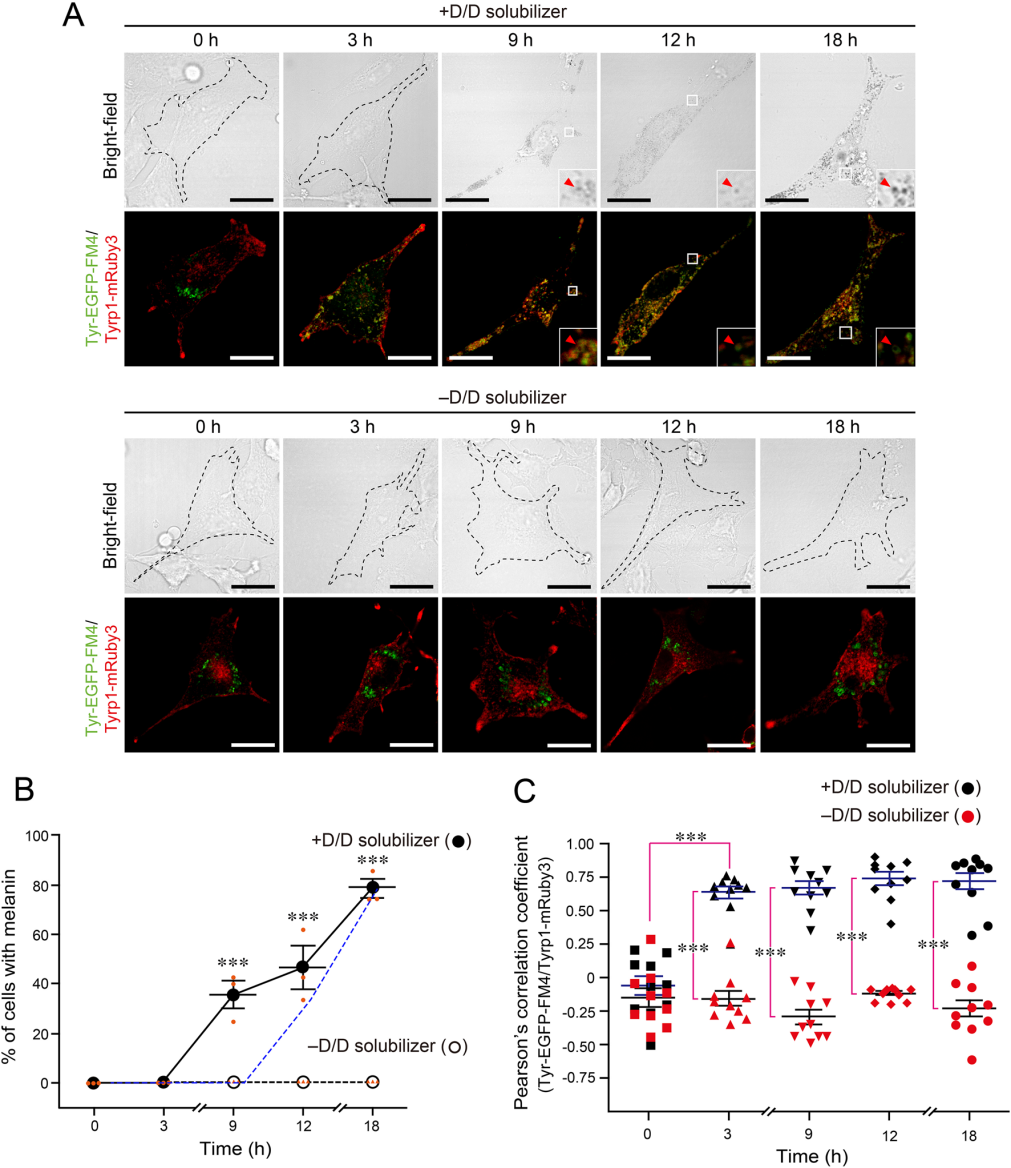
Overexpression of Tyrp1 promoted Tyr-mediated melanin synthesis. (**A**) mRuby3-tagged Tyrp1 and Tyr-EGFP-FM4 were co-expressed in *Tyr*-KO B16-F1 cells, and the cells were treated with D/D solubilizer or DMSO (–D/D solubilizer) and fixed at the times indicated. Representative images are shown. Transparent Tyr-EGFP-FM4-expressing cells in the bright-field images are outlined with broken black lines. Scale bars, 20 μm. (**B**) The percentage of cells shown in (**A**) containing melanin after treatment with D/D solubilizer (closed circles) or DMSO (–D/D solubilizer; open circles) was calculated. Blue broken lines indicate the melanin-containing cells expressing Tyr-EGFP-FM4 alone (see Fig. 2D). The error bars represent the means ± SEM of the data obtained in three independent experiments (n = 10 cells in each experiment). ∗ ∗ ∗ *P* < 0.001 (one-way ANOVA and Tukey’s test). (**C**) Quantification of the colocalization ratio between Tyr-EGFP-FM4 and Tyrp1-mRuby3 in the cells shown in (**A**) in the presence (black symbols) or absence of D/D solubilizer (red symbols). Pearson’s correlation coefficients were calculated (n = 10 cells), and the error bars represent the means ± SEM. ∗ ∗ ∗ *P* < 0.001 (one-way ANOVA and Tukey’s test).

We then used fluorescently tagged Tyrp1 (i.e., Tyrp1-monomeric Ruby3 [mRuby3]) to overcome the problem of the loss of Tyrp1 signals after melanin deposition in melanosomes and to visualize Tyr-deficient immature melanosomes in living cells. When Tyrp1-mRuby3 and Tyr-EGFP-FM4 were co-expressed in *Tyr*-KO cells, Tyrp1-mRuby3 was distributed in the periphery, the same as endogenous Tyrp1 (Fig. 3C), and no colocalization between Tyr-EGFP-FM4 and Tyrp1 was observed in the absence of D/D solubilizer (Fig. 4A, lower panels). By contrast, Tyr-EGFP-FM4 was transported to the peripheral area and colocalized with Tyrp1 there at 3 h after D/D solubilizer treatment, and melanin was observed at 9 h (Fig. 4A, upper panels). The results of the quantitative analysis of the melanin-containing cells revealed that ~ 40% of the Tyr-EGFP-FM4-expressing cells contained melanin at 9 h after the D/D solubilizer treatment, and ~ 80% of the cells were recovered at 18 h (Fig. 4B). Quantification of the colocalization rate between Tyr-EGFP-FM4 and Tyrp1-mRuby3 in the cells shown in Fig. 4A showed significant colocalization at 3 h after D/D solubilizer treatment (Fig. 4C). These results were somewhat unexpected, because colocalization between Tyr and Tyrp1 (a melanosome marker) and melanin synthesis in Tyrp1-mRuby3-expressing cells was observed ~ 6 h earlier than it was in untransfected cells (compare the solid line and broken blue line in Fig. 4B, and compare Figs. 3D and 4C). We therefore hypothesized that Tyrp1 plays a role in efficient expression and/or transport of Tyr to melanosomes in addition to its role in melanin synthesis. To test our hypothesis, we knocked down endogenous Tyrp1 in *Tyr*-KO cells and evaluated the effect of its knockdown on Tyr-EGFP-FM4 localization and melanin synthesis.

### Knockdown of endogenous Tyrp1 delayed Tyr-mediated melanin synthesis

**Figure 5:**
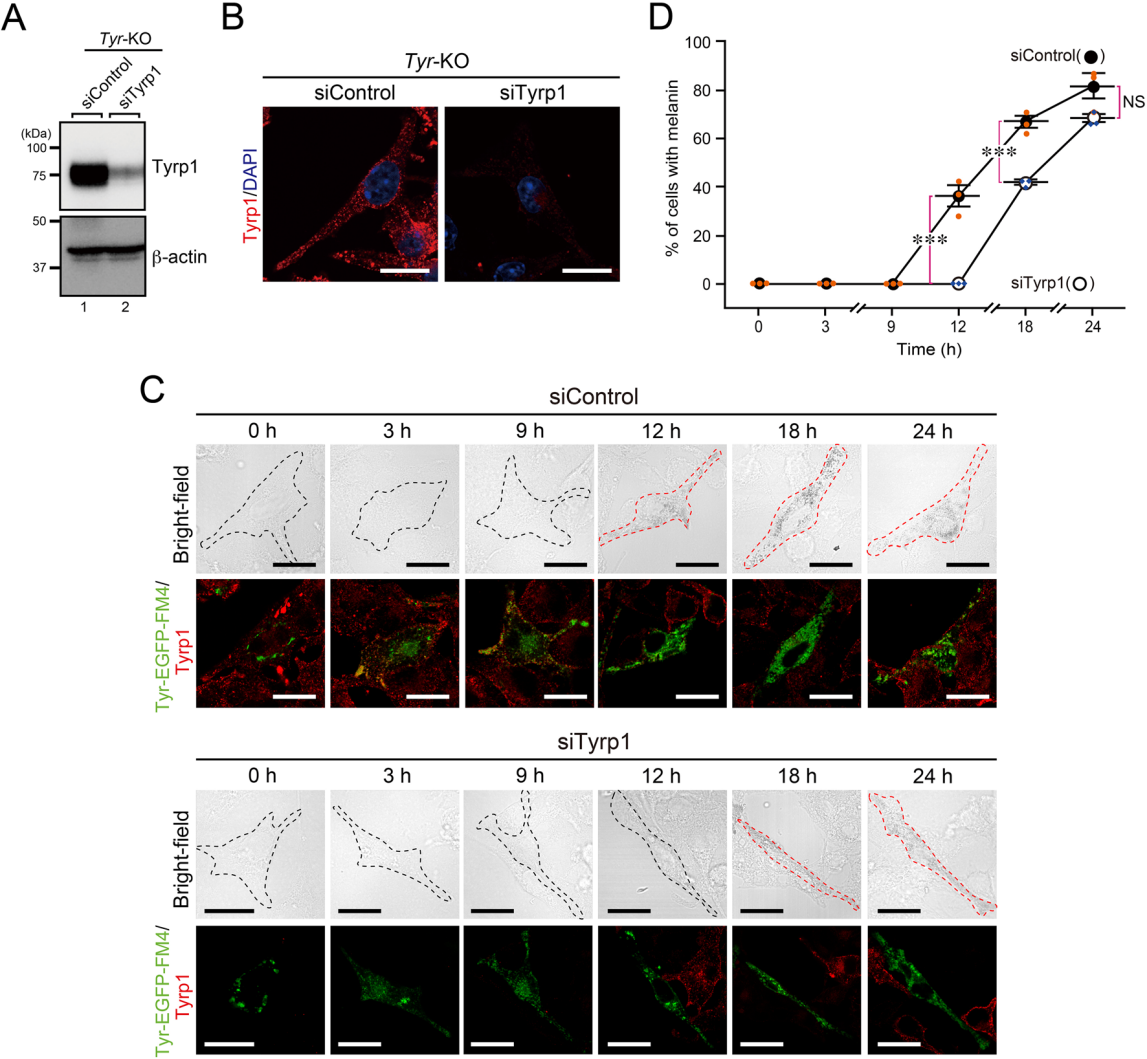
Knockdown of Tyrp1 delayed Tyr-mediated melanin synthesis. (**A,B**) Knockdown efficiency of Tyrp1 in *Tyr*-KO B16-F1 cells transfected with siRNA against Tyrp1 (siTyrp1) or control siRNA. The cells were analyzed by immunoblotting with anti-Tyrp1 and anti-β-actin antibodies (**A**) and by immunofluorescence using anti-Tyrp1 antibody (**B**). Scale bars, 20 μm. (**C**) Tyrp1-depleted (or control) Tyr-EGFP-FM4-expressing *Tyr*-KO cells were treated with D/D solubilizer and fixed at the times indicated. Melanin-containing Tyr-EGFP-FM4-expressing cells and transparent Tyr-EGFP-FM4-expressing cells in the bright-field images are outlined with broken red lines and broken black lines, respectively. Scale bars, 20 μm. (**D**) The percentage of cells shown in (**C**) containing melanin after treatment with D/D solubilizer was calculated. Tyrp1-depleted and controls cells are shown by closed circles and open circles, respectively. The error bars represent the means ± SEM of the data obtained in three independent experiments (n = 10 cells in each experiment). ∗ ∗ ∗ *P* < 0.001; NS, not significant (one-way ANOVA and Tukey’s test).

We used a specific siRNA against Tyrp1 (siTyrp1) to knock down endogenous Tyrp1 in *Tyr*-KO cells and confirmed its depletion by immunoblotting and immunofluorescence analysis (Fig. 5A,B). We then performed the same Tyr-EGFP-FM4 synchronized transport assay shown in Fig. 2 to evaluate the recovery of black melanosomes (i.e., melanin) under the Tyrp1-depleted conditions. The results showed a significant delay in the appearance of black melanosomes in the Tyrp1-depleted cells in comparison with the control siRNA (siControl)-treated cells (Fig. 5C,D). Melanin was first observed in the Tyrp1-depleted cells at 18 h after the D/D solubilizer treatment (Fig. 5C, lower panels), which was significantly later than in the control cells (Fig. 5C, upper panels, 12 h). These results taken together demonstrated that Tyrp1 is a positive regulator of efficient targeting of Tyr to melanosomes as well as of efficient melanin synthesis.

## Discussion

In the present study, we succeeded in establishing a synchronized Tyr transport system by using Tyr-EGFP-FM4 in *Tyr*-KO cells. Tyr-EGFP-FM4 transport was initiated upon D/D solubilizer treatment, and it was first transported to the Golgi at 30 min (Fig. 3B), the same as other secretory proteins^22,23^, and then reached Tyrp1-positive immature melanosomes at 9 h (Fig. 3D). There was a time lag (~ 3 h) between the melanosome localization of Tyr-EGFP-FM4 and the appearance of black mature melanosomes (Figs. 2D and 3D). Using the synchronized Tyr transport system also unexpectedly revealed that Tyrp1 is involved in efficient melanosomal localization of Tyr and melanin synthesis (Figs. 4 and 5). How does Tyrp1 regulate Tyr transport and Tyr-mediated melanin synthesis? There had been reports that Tyrp1 and Dct (dopachrome tautomerase; also called Tyrp2) modulate Tyr activity and stabilize the Tyr protein through heterooligomer formation^25–28^, and consistent with these reports, we observed the Tyr–Tyrp1 interaction only in the presence of D/D solubilizer (Fig. S2). Since the Tyr and Tyrp1 transport mechanisms appeared to be somewhat different, e.g., adaptor complex-1 (AP-1) and AP-3 differentially control the transport of Tyr and the transport of Tyrp1, respectively^29,30^, a possible advantage of the heterodimer formation would be the use of a different transport mechanism that a monomer Tyr or Tyrp1 would not usually be able to use. Thus, the Tyr–Tyrp1 heterodimer may be transported to immature melanosomes more efficiently than a Tyr or Tyrp1 monomer. Alternatively, Tyrp1 may simply be involved in the proper folding and stabilization of Tyr protein in the ER, thereby enabling its efficient targeting to melanosomes. Further extensive research will be needed to pursue these possibilities.

Melanogenic enzymes, including Tyr, are generally thought to be transported to melanosomes via endosomes, and several gene products responsible for genetic pigmentation disorders, e.g., BLOC complexes and certain Rabs, are actually involved in endosomal transport^8–10^. However, the precise roles of these gene products in each of the Tyr transport events (e.g., ER-to-Golgi transport, transport from the Golgi, and endosomal transport) are not fully understood, and somewhat conflicting results have been reported. For example, Rab32/38 and their regulators (e.g., the Rab32/38 effector Varp and their activator BLOC-3) were initially reported to regulate the post-Golgi transport of Tyr to immature melanosomes^15,16,31^, but a subsequent study showed that they regulate the recycling of VAMP7, which presumably mediates the fusion of Tyr-containing vesicles with immature melanosomes^32,33^, from melanosomes^17^. Thus, the synchronized Tyr transport system that we established in this study should provide an ideal tool to determine whether Rab32/38 are involved in Tyr transport to immature melanosomes, VAMP7 recycling from melanosomes, or both by using appropriate endosome markers in combination with live-cell imaging. It will also be possible to evaluate the significance of post-translational modifications such as palmitoylation of Tyr^34^ and ubiquitylation^35,36^ in Tyr transport by using Tyr-EGFP-FM4 mutants lacking post-translational modification sites. In addition to using the synchronized Tyr transport system to re-assess or re-investigate known regulators or modifications of Tyr, it can be applied to the search for new Tyr transport regulators by performing siRNA screening or CRISPR/Cas9-mediated genome-wide screening in the future. Moreover, the synchronized Tyr transport system will be useful for developing novel cosmetics (or drugs) for skin whitening (or for preventing grey hair) that can specifically inhibit (or promote) the Tyr transport process without affecting its enzymatic activity.

In summary, we succeeded in establishing a synchronized Tyr transport system by using Tyr-EGFP-FM4 in *Tyr*-KO cells, and it allowed us to analyze Tyr transport by 30 min of intervals and to identify Tyrp1 as a positive regulator of efficient Tyr transport to melanosomes. In the future, the tool we established in this study should be useful in basic science studies designed to understand the precise molecular mechanism of Tyr transport in melanocytes as well as in industry for research to develop new drugs or cosmetics that artificially regulate Tyr transport.

## Materials and methods

### Materials

The oligonucleotides, plasmids, and antibodies used in this study are summarized in Table S1. The mouse Tyrp1 cDNA was obtained by PCR as described previously^20^. The Tyr and FM4 cDNAs were also prepared as described previously^20,37^. The newly constructed plasmids, including pEF-Tyr-EGFP-FM4, used in this study were prepared by the standard molecular biology techniques. The Tyr-EGFP-FM4 and Tyrp1-mRuby3 expression plasmids are available from RIKEN BioResource Research Center in Japan (https://dnaconda.riken.jp/search/depositor/dep005893.html; Cat# RDB20230 and RDB20231, respectively). Unless otherwise specified, all other general reagents used in this study were analytical grade or the highest grade commercially available.

### Cell cultures and transfections

The B16-F1 melanoma cells (obtained from the American Type Culture Collection, Manassas, VA, USA), *Tyr*-KO B16-F1 cells^21^, and COS-7 cells (obtained from RIKEN BioResource Research Center, Tsukuba, Japan) were cultured at 37 °C under 5% CO_2_ in D-MEM medium (FUJIFILM Wako Pure Chemical, Osaka, Japan) containing 10% fetal bovine serum, 100 units/mL penicillin G, and 100 µg/mL streptomycin. In the KD experiments, B16-F1 cells were transfected with siRNAs (final concentration 100 nM) by using RNAiMAX (Thermo Fisher Scientific, Waltham, MA, USA) according to the manufacturer’s instructions, and then cultured for 48 h. In the immunofluorescence analysis, B16-F1 cells were transfected with plasmid DNAs by using Lipofectamine 2000 (Thermo Fisher Scientific) according to the manufacturer’s instructions, and then cultured for 24–72 h. In the Tyr-EGFP-FM4 synchronized transport assays, cells were treated with 500 nM D/D solubilizer (Takara Bio, Shiga, Japan) at 24 h after transfection.

### Immunoblotting

Cells were lysed with 1 × SDS sample buffer and boiled for 10 min. The samples were separated by 7.5% or 10% SDS-PAGE and transferred to PVDF membranes (Merck Millipore, Burlington, MA, USA) by electroblotting using the Trans-Blot Turbo™ Transfer System (Bio-Rad, Hercules, CA, USA). After blocking the membranes for 30 min at room temperature with 1% skim milk in PBS containing 0.1% Tween-20 (PBS-T), they were incubated for 1 h at room temperature with primary antibodies diluted in 1% skim milk (antibody dilutions are summarized in Table S1). After washing the membranes with PBS-T three times, they were incubated for 1 h at room temperature with appropriate horseradish peroxidase (HRP)-conjugated secondary antibodies diluted in 1% skim milk. Immunoreactive bands were detected by using blotting detection reagents (Clarity™ Western ECL Substrate, Bio-Rad) and the chemiluminescence imager (ChemiDoc Touch, Bio-Rad). The blots shown in this study are representative of the blots obtained in three independent experiments (Fig. S3).

### Immunofluorescence analysis

*Tyr*-KO B16-F1 cells were fixed with 4% paraformaldehyde for 10 min, permeabilized with 0.05% saponin for 30 min, and blocked with 1% bovine serum albumin in PBS for 30 min. The cells were stained with specific primary antibodies (antibody dilutions are summarized in Table S1) and then visualized with Alexa Fluor 488/555-conjugated secondary antibodies. The stained cells were examined for fluorescence with a confocal fluorescence microscope (FluoView 1000-D, Evident/Olympus, Tokyo, Japan) through a 60 × objective lens (numerical aperture 1.40; Evident/Olympus) and with the FluoView software (version 4.1a, Evident/Olympus). The fluorescence images and their corresponding bright-field images were captured at random with the confocal microscope and quantified with ImageJ software (version 1.52i; National Institutes of Health, Bethesda, MD, USA).

### Co-immunoprecipitation assay

COS-7 cells were co-transfected with pEF-Tyr-EGFP-FM4 and pMRX-bsr-Tyrp1-monomeric Strawberry (mStr) and incubated for 9 h. The cells were treated with 500 nM D/D solubilizer for 24 h and then collected and lysed for 1 h on ice in a lysis buffer (50 mM HEPES–KOH, pH 7.2, 150 mM NaCl, and 1% Triton X-100 supplemented with a complete EDTA-free protease inhibitor cocktail [Roche, Basel, Switzerland]). After centrifugation at 700 × *g* for 10 min at 4 °C to remove insoluble materials, the supernatants were incubated with gentle rotation for 1 h at 4 °C with GST-fused green fluorescent protein (GFP) nanobody^38^-coupled with glutathione Sepharose™ 4B (GE Healthcare, Chicago, IL, USA). After washing the beads with the lysis buffer three times, they were suspended in 50 µL of 1 × SDS sample buffer and boiled for 10 min, followed by immunoblotting analyses.

### Statistical analysis

The statistical analyses were performed by one-way ANOVA followed by Tukey’s test with GraphPad Prism 9 Software (GraphPad Software, Inc, La Jolla, CA, USA). All quantitative data are expressed as the means ± SEM. The asterisks in the graphs indicate *P* values (∗*P* < 0.05; ∗∗*P* < 0.01; and ∗∗∗*P* < 0.001). NS stand for not significant (*P* > 0.05).

### Supplementary Information

Supplementary Information.

## Data Availability

The datasets used and/or analyzed during the current study available from the corresponding author on reasonable request.
