# Supplementary material for: Establishment of a synchronized tyrosinase transport system revealed a role of Tyrp1 in efficient melanogenesis by promoting tyrosinase targeting to melanosomes
Source: Sci Rep. 2024 Jan 30;14:2529. doi: 10.1038/s41598-024-53072-6 (PMC10827793; doi:10.1038/s41598-024-53072-6)
Supplement: Supplementary file 1 — Supplementary Information. [file 41598_2024_53072_MOESM1_ESM.pdf]

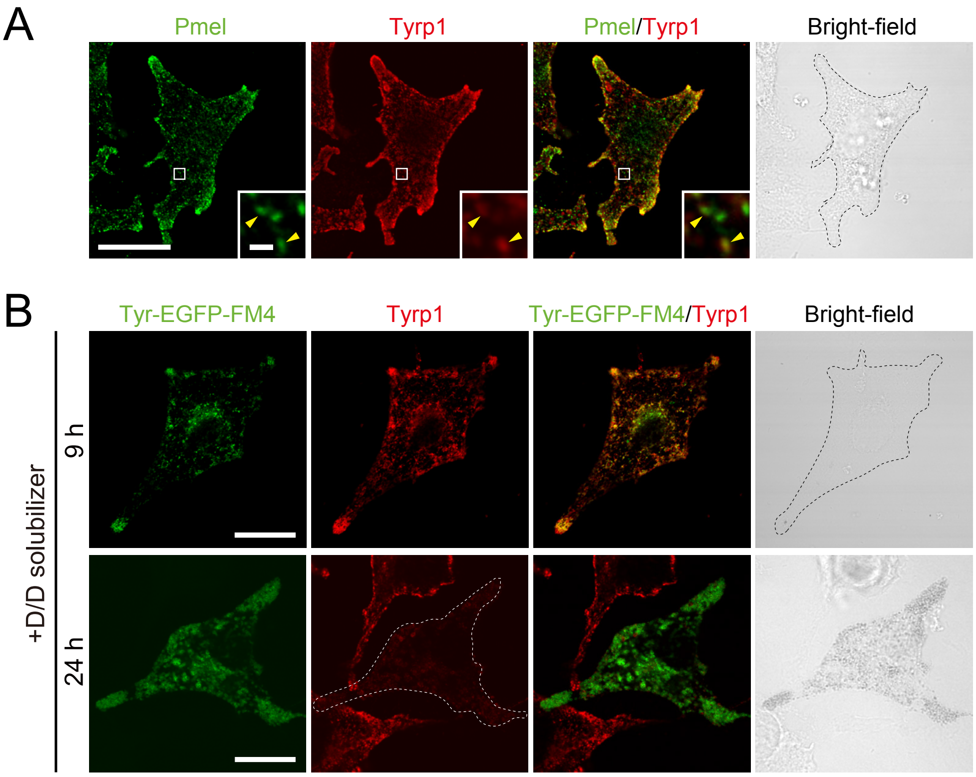

Figure S1. Additional data on Tyrp1 in *Tyr*-KO B16-F1 cells. (**A**) Colocalization between Tyrp1 and Pmel in *Tyr*-KO cells. The cells were fixed and stained for Tyrp1 (red) and Pmel (green). The arrowheads in the insets show the colocalization points. Scale bars, 20 µm (1 µm in the insets). (**B**) Deficient Tyrp1 signals in melanin-containing *Tyr*-KO cells expressing Tyr-EGFP-FM4. The cells were fixed at 9 h or 12 h after treatment with D/D solubilizer and stained for Tyrp1 (red). Note the clear presence of Tyrp1 signals in the transparent cells (upper panels) and their absence in the melanin-containing cells (outlined with broken white lines; lower panels). Scale bars, 20 µm.

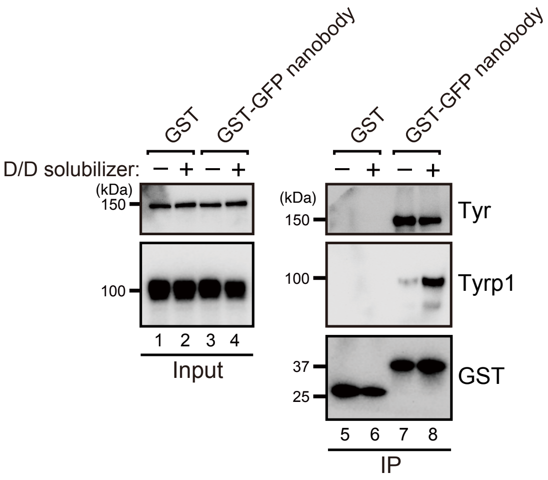

Figure S2. D/D solubilizer-dependent interaction between Tyr and Tyrp1. Tyr-EGFP-FM4 and Tyrp1-mStr were co-expressed in COS-7 cells, and the cells were treated for 9 h with D/D solubilizer or DMSO (–D/D solubilizer). The interaction between Tyr and Tyrp1 was evaluated by co-immunoprecipitation (IP) assays performed with beads coupled with GST-GFP-nanobody. GST alone was used as a negative control. Proteins bound to the beads were analyzed by immunoblotting with the antibodies indicated. Note the clear Tyr–Tyrp1 interaction only in the presence of D/D solubilizer (lane 8).

A

Fig. 1B - top panel

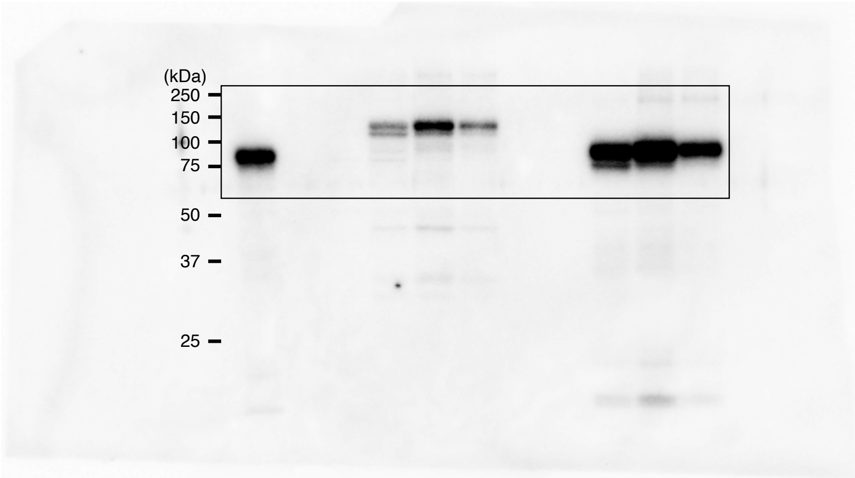

Fig. 1B - bottom panel

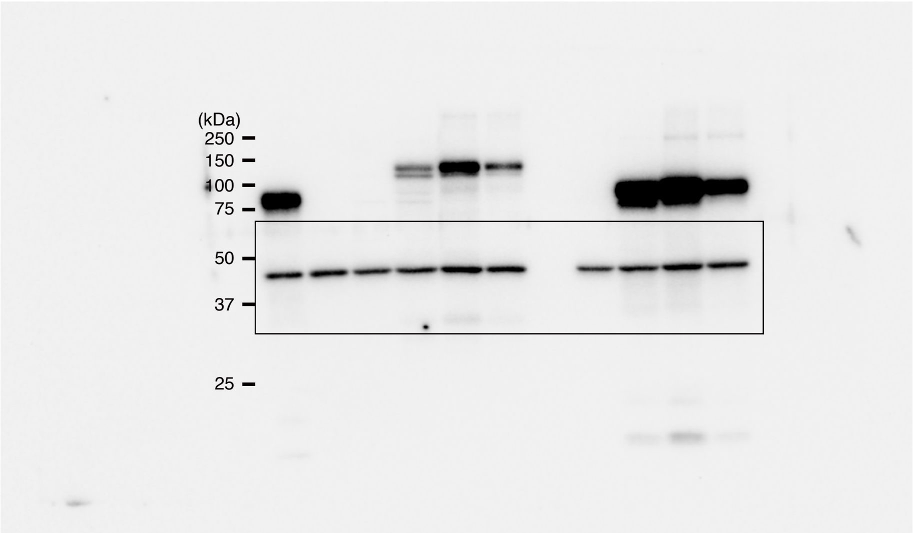

B

Fig. 2B- top panel

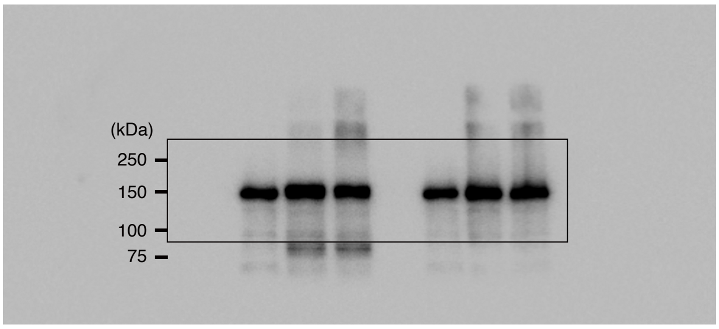

Fig. 2B - bottom panel

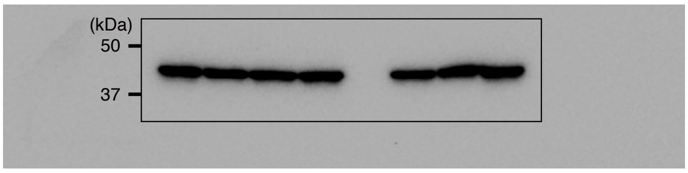

C

Fig. 5A - top panel

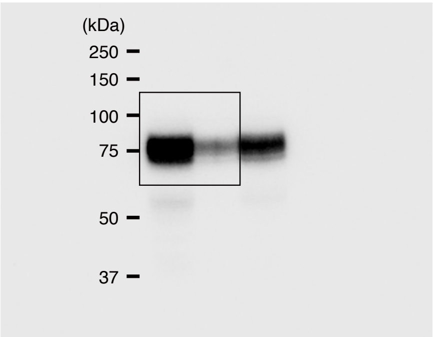

Fig. 5A - bottom panel

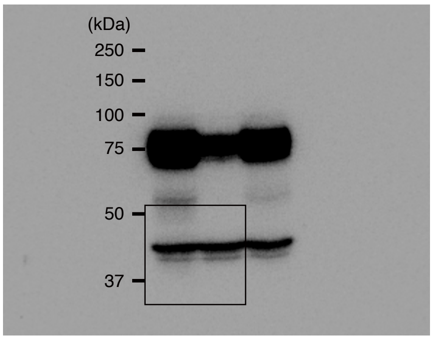

D

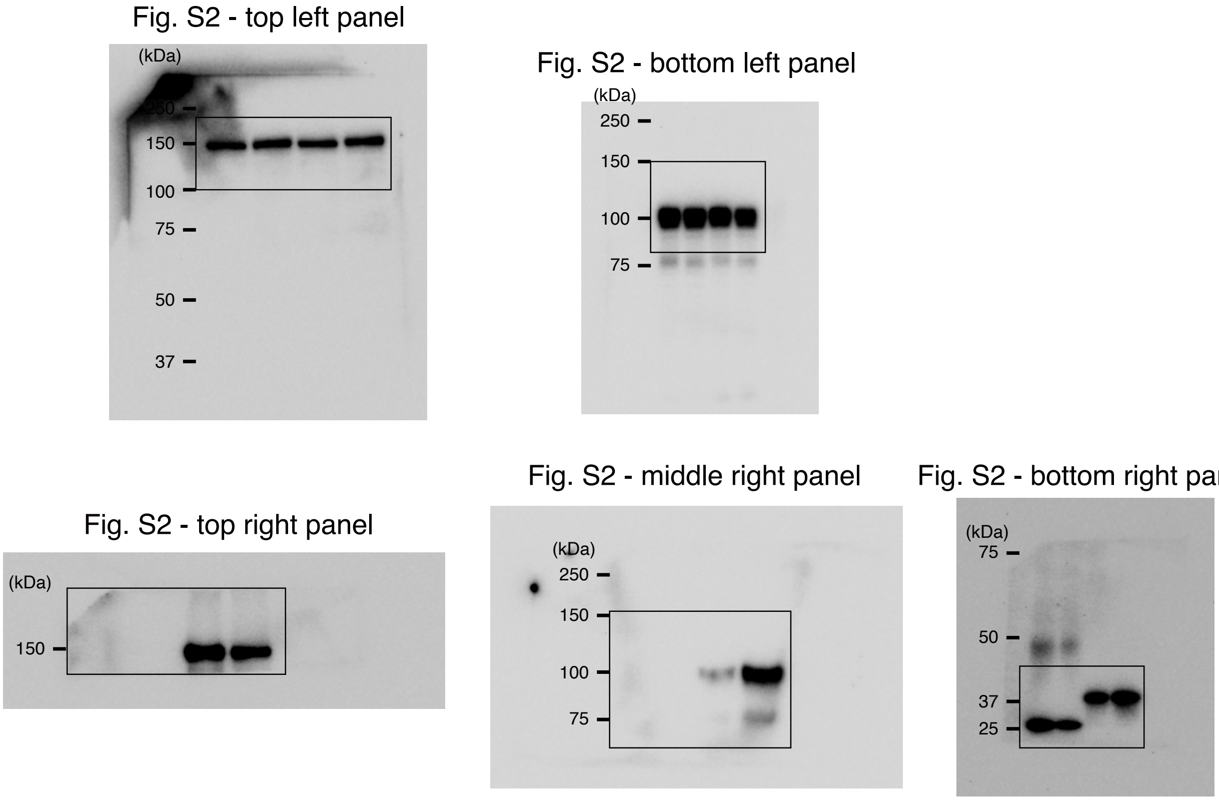

Figure S3. Original data on immunoblots used in this study. (**A**) Original blot data shown in Fig. 1B. (**B**) Original blot data shown in Fig. 2B. (**C**) Original blot data shown in Fig. 5A. (**D**) Original blot data shown in Fig. S3.

Table S1. List of materials used in this study

Plasmids	RIKEN BioResource Research Center Cat#	Source
pEF-Tyr-EGFP		This study
pEF-Tyr-EGFP-FM4	RDB20230	This study
pEF-Tyr-FLAG	RDB14635	*J. Biochem.* (2017) **161**, 323–326
pEF-Tyrp1-mRuby3	RDB20231	This study
pMRX-bsr-Tyrp1-mStr		This study

Antibodies	Dilution	Source
anti-Tyr rabbit pAb	IB (0.27 μg/mL)	*Traffic* (20119 **12**, 627–643
anti-Tyr mouse mAb (4H12-9F12)	IF (1/5,000 dilution)	This study (produced in COSMO Bio, Tokyo, Japan)
anti-GM130 mouse mAb	IF (1/5,000 dilution)	BD Biosciences (Franklin Lakes, NJ) 610822
anti-Tyrp1mouse mAb (TA99)	IF (1/5,000 dilution)	Santa Cruz Biotechnology (Dallas, TX) sc-58438
anti-Tyrp1 rabbit pAb	IB (0.228 μg/mL)	*J. Invest. Dermatol.* (2013) **133**, 2237–2246
anti-Pmel mouse mAb (HMB45)	IF (1/2,000 dilution)	DAKO (Amsterdam, Netherlands) M0634
anti-RFP pAb	IB (1/5,000 dilution)	MBL (Tokyo Japan) PM005
anti-β-actin-HRP mouse mAb	IB (1/5,000 dilution)	Proteintech (Rosemont, IL) HRP-60008
anti-GST mouse mAb (B-14)	IB (1/5,000 dilution)	Santa Cruz Biotechnology (Dallas, TX) sc-138

siRNA	Target sequence	Source
mouse Tyrp1 siRNA	ACATGTCAGTGCAATGATA	Nippon Gene (Toyama, Japan)
control siRNA (Luc GL2)	CGTACGCGGAATACTTCGA	Nippon Gene (Toyama, Japan)

IB, immunoblot; IF, immunofluorescence; mAb, monoclonal antibody; pAb, polyclonal antibody.
